# Evaluation of the Perioperative and Postoperative Course of Surgery for Pineal Germinoma in the SIOP CNS GCT 96 Trial

**DOI:** 10.3390/cancers14143555

**Published:** 2022-07-21

**Authors:** Ehab Shabo, Thomas Czech, James C. Nicholson, Conor Mallucci, Carmine Mottolese, Gianluca Piatelli, Didier Frappaz, Matthew Jonathan Murray, Cecile Faure-Conter, Maria Luisa Garrè, Sevgi Sarikaya-Seiwert, Leonie Weinhold, Hannes Haberl, Gabriele Calaminus

**Affiliations:** 1 Department of Neurosurgery, Rheinische Friedrich-Wilhelms-University, Sigmund-Freud-Straße 25, 53127 Bonn, Germany; 2 Department of Neurosurgery, Medical University of Vienna, 1090 Vienna, Austria; thomas.czech@meduniwien.ac.at; 3 Department of Paediatric Haematology and Oncology, Cambridge University Hospitals NHS Foundation Trust, Cambridge CB2 0QQ, UK; james.nicholson@addenbrookes.nhs.uk; 4 Department of Neurosurgery, Alder Hey Children’s Hospital, Liverpool L14 5AB, UK; conor.mallucci@alderhey.nhs.uk (C.M.); mjm16@cam.ac.uk (M.J.M.); 5 Neurochirurgie Pédiatrique, Hôpital Femme-Mère-Enfant, Université de Lyon, 59, Boulevard Pinel, 69003 Lyon, France; carmine.mottolese@chu-lyon.fr; 6 Division of Neurosurgery, Department of Neurosciences, Giannina Gaslini Children’s Hospital, 16147 Genvoa, Italy; gianlucapiatelli@gaslini.org; 7 Department of Pediatric Hematology and Oncology, Institut d’Hématologie et d’Oncologie Pédiatrique, 69008 Lyon, France; didier.frappaz@ihope.fr (D.F.); cecile.conter@ihope.fr (C.F.-C.); 8 Department of Pathology, University of Cambridge, Tennis Court Road, Cambridge CB2 1QP, UK; 9 Department of Pediatric Hematology-Oncology and Bone Marrow Transplantation, IRCCS Istituto Giannina Gaslini, 16147 Genova, Italy; mluisagarre@gaslini.org; 10 Section of Pediatric Neurosurgery, Department of Neurosurgery, Rheinische, Friedrich-Wilhelms-University, Sigmund-Freud-Straße 25, 53127 Bonn, Germany; sevgi.sarikaya-seiwert@ukbonn.de (S.S.-S.); hhaberl@aol.com (H.H.); 11 Department of Medical Biometry, Informatics and Epidemiology, University Hospital Bonn, 53127 Bonn, Germany; leonie.weinhold@ukbonn.de; 12 Department of Pediatric Hematology and Oncology, University Hospital Bonn, Venusberg-Campus 1, 53127 Bonn, Germany; gabriele.calaminus@ukbonn.de

**Keywords:** pineal germinoma, resection, biopsy, perioperative course, SIOP CNS GCT 96 trial

## Abstract

**Simple Summary:**

There are many studies that discuss pineal tumors (not specifically germinoma). Some advocate for biopsy over resection due to the fact that germinoma is radio- and chemosensitive; others advocate for primary resection due to multiple differential diagnoses that require resection in the pineal region, such as pineocytoma, teratoma, meningioma, epidermoid, etc. However, with respect to solitary pineal germinoma, there is still no study that analyzes and compares both surgical options regarding peri- and postoperative courses and complications. We evaluated the perioperative course and postoperative complications of patients with isolated pineal germinoma who underwent a primary biopsy or resection, treated according to the European SIOP CNS GCT 96 trial. The key finding of our study was that postoperative complications, including hydrocephalus, Parinaud syndrome, double vision, etc., were significantly higher in patients who underwent primary resection (*p* < 0.008).

**Abstract:**

Background: CNS germinoma, being marker-negative, are mainly diagnosed by histological examination. These tumors predominantly appear in the suprasellar and/or pineal region. In contrast to the suprasellar region, where biopsy is the standard procedure in case of a suspected germ-cell tumor to avoid mutilation to the endocrine structures, pineal tumors are more accessible to primary resection. We evaluated the perioperative course of patients with pineal germinoma who were diagnosed by primary biopsy or resection in the SIOP CNS GCT 96 trial. Methods: Overall, 235 patients had germinoma, with pineal localization in 113. The relationship between initial symptoms, tumor size, and postoperative complications was analyzed. Results: Of 111 evaluable patients, initial symptoms were headache (*n* = 98), hydrocephalus (*n* = 93), double vision (*n* = 62), Parinaud syndrome (*n* = 57), and papilledema (*n* = 44). There was no significant relationship between tumor size and primary symptoms. A total of 57 patients underwent primary resection and 54 underwent biopsy. Postoperative complications were reported in 43.2% of patients after resection and in 11.4% after biopsy (*p* < 0.008). Biopsy was significantly more commonly performed on larger tumors (*p*= 0.002). Conclusions: These results support the practice of biopsy over resection for histological confirmation of pineal germinoma.

## 1. Introduction

Intracranial germ cell tumors (GCTs) represent about 0.5 to 3 percent of pediatric central nervous system (CNS) tumors in North America and Europe. By contrast, these tumors are substantially more frequent in Japan and other Asian countries, with a reported incidence of up to 11 percent of pediatric CNS tumors [1]. The peak incidence of intracranial GCT is during the second decade of life, with a median age at diagnosis of 10 to 14 years [2]. There is a male preponderance of between 2:1 to 3:1, especially with tumors in the pineal region [3,4]. Intracranial GCTs arise almost exclusively from midline locations. The two most frequent sites are the pineal and suprasellar regions, with pineal tumors occurring nearly twice as often as suprasellar GCTs [5,6].

GCTs are the most common type of pineal gland tumors [7]. In the literature, the incidence of GCTs varies from 50 to 75% of tumors in the pineal region [1,8,9]. GCTs are further classified into two groups: germinomatous, which is the most common subtype, and nongerminomatous (NGGCTs). Pineal parenchymal tumors are the second most common form of pineal tumors. They represent 14–27% of tumors in the pineal gland [10]. Other CNS tumors can arise from the supporting stroma of the pineal gland and adjacent structures. These tumors include low- and high-grade gliomas, ependymoma, embryonal tumors, pineal parenchymal tumors, and papillary tumors of the pineal region [10,11].

Currently, advanced neuroimaging can provide information to differentiate these entities based on growth pattern and tissue characteristics prior to any intervention, thereby supporting the clinician in deciding on a treatment plan.

Intracranial germinoma are exquisitely radio- and chemosensitive [12,13,14,15,16,17,18,19,20,21,22,23,24,25,26]. However, in the European protocols (SIOP CNS GCT 96 and SIOP CNS GCTII), surgery to obtain tissue for diagnosis is mandatory for patients with tumor markers below defined thresholds. In Europe, such ‘marker-negative’ cases are defined by alpha-fetoprotein (AFP) levels below 25 ng/mL and human chorionic gonadotropin (hCG) levels below 50 UI/L, both in the cerebrospinal fluid (CSF) and in the serum. This approach allows pure germinoma or mature teratoma to be distinguished from other benign and malignant lesions.

Of note, the role of neurosurgery (resection versus biopsy) for patients with intracranial GCTs in the pineal region, and its complications, have not yet been thoroughly investigated in large cohorts. Contemporary series suggest that stereotactic biopsy is reasonably safe and well-tolerated [27,28,29,30]. Histology obtained by stereotactic biopsy is informative in 94 to 100 percent of cases when multiple target biopsies are obtained, but sampling error may be an issue due to the heterogeneity of pineal GCTs, particularly with mixed histology [31]. Intervention-related morbidity is generally limited to transient worsening of ocular symptoms, although fatal complications have also been reported [28]. A frameless stereotactic robot has also been successfully used for brain biopsies over the past few years [32].

Open surgery rather than stereotactic biopsy, including the option at an attempt for a gross total resection, is favored by some experts as the initial approach to patients with pineal tumors. Because approximately one-third of pineal lesions are benign, open surgery aiming for complete resection of the lesion may be potentially curative as well as diagnostic [33,34,35,36]. Furthermore, an open procedure minimizes sampling error, and tumor debulking may obviate the need for CSF diversion if obstructive hydrocephalus is present [36,37]. However, an attempt for a gross total resection for CNS GCTs is not widely accepted for several reasons. The risk of lasting morbidity from gross total resection is 3 to 10 percent [33,34], while mortality rates range from 4 to 10 percent [38], whereas mortality and morbidity rates for stereotactic biopsy of the pineal region range between 1% and 1.3% [28]. The endoscopic approach with biopsy, also under visual control, in addition to allowing for CSF-sampling, permits direct inspection of parts of the ventricular walls for staging purposes, and allows a third ventriculostomy to be performed for CSF diversion if needed [39].

These aspects and an increased familiarity with neuroendoscopic techniques have led to a shift toward an endoscopic approach for the biopsy of these lesions [40,41,42]. Any potential benefit from an attempt at an open resection must be balanced against procedural risks.

Although current consensus is to rely on biopsy to diagnose intracranial germinoma due to their high radio- and chemosensitivity, limited systematic and statistical evidence has been provided to support this strategy especially in respect of solitary pineal germinoma [36]. The purpose of this report is to evaluate and compare the perioperative course as well as the postoperative complications of initial primary surgical interventions, namely resection versus biopsy, observed in a large number of patients with solitary pineal germinoma in the international SIOP CNS GCT 96 trial. The evolution of the surgical approach in the presence of a marker-negative pineal mass suggestive of a GCT over the study period was also evaluated.

## 2. Materials and Methods

Patients’ data were obtained from the nonrandomized international SIOP CNS GCT 96 study. A total of 235 patients (176 men, 59 women) with a histologically confirmed diagnosis of a germinoma were enrolled in SIOP CNS GCT 96 from 1 January 1996 through 31 December 2005, and followed up to 18 July 2012 [43].

**Figure 1:**
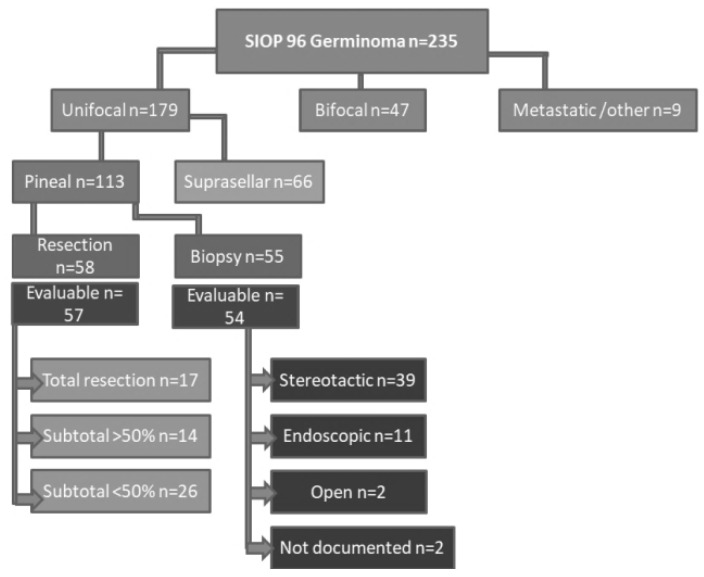
SIOP GCT CNS 96 study patients and classification of two groups (resection and biopsy).

Of the total 235 patients, 179 had a unifocal germinoma, 113 of which were localized in the pineal region, and the other 66 in the suprasellar region. Forty-seven patients were diagnosed with bifocal and nine patients with metastatic germinomas (Figure 1).

Concerning the contribution of the countries in respect of pineal germinoma, patients were registered from 14 countries. Most patients were registered from Germany *n* = 32, followed by France *n* = 20, the United Kingdom *n* = 17, Austria *n* = 8, Switzerland *n* = 7, the Netherlands *n* = 6, Poland *n* = 6, Belgium *n* = 4, Denmark *n* = 3, Greece *n* = 3, Italy *n* = 3, Norway *n* = 2, Spain *n* = 1, and Sweden *n* = 1 (total of 113 pineal germinoma).

In this study, we enrolled all 113 patients with an isolated pineal gland germinoma and divided them into 2 groups: Group 1 (resection group) included 58 patients who underwent a primary resection, and Group 2 (biopsy group) included 55 patients who were initially biopsied.

After reviewing the SIOP CNS GCT 96 database, 57 patients in Group 1 (53 men, 4 women) and 54 patients in Group 2 (53 men, 1 women) were evaluable. The remaining two patients were not evaluable due to incomplete surgical and perioperative documentation. The median age was 14 years (range, 7–42 years).

In Group 1, the extent of resection (EOR) was categorized based on the intraoperative impression of the surgeon combined with results of postoperative imaging by CT or MRI as total resection, subtotal resection (>50%) and partial resection (<50%).

Group 2 was divided into 3 subgroups based on the surgical biopsy technique used (stereotactic, endoscopic, and open biopsy). Initial clinical signs and symptoms, tumor size defined as the largest tumor diameter in any plane, type of surgery, postoperative complications, and pre- and postoperative performance status as assessed by the Lansky/Karnofsky scale were analyzed.

The characteristics of the study patients are described as median values (with range) or numbers with percentages as appropriate. The relation between group and tumor size (categorized in SIOP CNS GCT 96 protocol in the radiological section in <2 cm diameter, 2–3 cm, >3 cm), between groups and primary symptoms and between groups and postoperative complications were assessed by the chi-square test or Fisher’s exact test where appropriate. Patients with incomplete documentation were excluded from the statistical analysis. Analysis was conducted using the IBM® SPSS® Statistics (version 25, IBM Corp., Armonk, NY) with *p*-values < 0.05 considered statistically significant.

## 3. Results

Of 111 evaluable patients (106 men, 5 women) with unifocal pineal germinoma, pure germinoma was present in 101 patients (91.0%) The other 10 (9.0%) had additional teratoma components: 6 (10.5%) patients were from the resection group and 4 (7.4%) from the biopsy group. No patients presented with other nongerminomatous components.

### 3.1. Group 1 (Resection Group)

We found that 57 patients had a debulking procedure with a total resection achieved in 17 (29.8%), a subtotal resection (>50%) in 14 (24.6%) and a partial resection (<50%) in 26 (45.6%) patients (Figure 1). The main primary symptoms in Group 1 were hydrocephalus-related (*n* = 50 (87.71%)), headache (*n* = 47 (82.45%)), Parinaud syndrome (*n* = 34 (59.64%)), double vision (*n* = 32 (56.14%)), and papilledema (*n* = 20 (35.08%)). The most common operative approach used was the supracerebellar infratentorial approach (*n* = 36 (63.2%)). Other approaches (e.g., occipital transtentorial (*n* = 2 [3.5%]), supratentorial transcortical-transventricular (*n* = 2 [3.5%]), and endoscopic approaches (*n* = 3 [5.3%])) were less frequently and individually practiced. The surgical approach was not mentioned or documented in 14 patients (24.6%).

### 3.2. Group 2 (Biopsy Group)

Biopsy-only was reported in 54 patients. Frame-based stereotaxy was used in 39 (72.2%) patients. An endoscopic biopsy was performed in 11 (20.4%) patients, and in 2 (3.7%) patients, biopsy was obtained by an open microsurgical approach. The remaining two patients underwent a biopsy without further information on the technique used (Figure 1).

The main primary symptoms in Group 2 were headache (*n* = 51 (94.4%)), hydrocephalus-related (*n* = 43 (79.6%)), double vision (*n* = 30 (55.6%)), papilledema (*n* = 24 (44.4%)), and Parinaud syndrome (*n* = 23 (42.6%)).

Considering both groups combined, the initial symptoms at diagnosis were headache (*n* = 98 (88.3%)), hydrocephalus-related (*n* = 93 (83.8%)), double vision (*n* = 62 (55.9%)), Parinaud syndrome (*n* = 57 (51.4%)), and papilledema (*n* = 44 (39.6%)). There was no significant difference between groups concerning primary symptoms (*p*-value > 0.05).

### 3.3. Tumor Size

The SIOP committee chose cut-off values for tumor size (<2 cm, 2–3c m, >3 cm diameter) in the SIOP CNS GCT 96 trial protocol in the radiological finding section and after the section of primary symptoms to evaluate the relationship between primary symptoms and tumor size.

Tumor size was documented in 81 patients (73.0%): 43 patients in Group 1 and 38 patients in Group 2. The tumor size in Group 1 was <2 cm diameter in 7 patients (16.3%), between 2 and 3 cm in 26 patients (60.5%), and >3 cm in 10 patients (23.2%). Notably, most of the patients in Group 2 had a tumor >3 cm (*n* = 22 (57.9%)): nine patients had a tumor size between 2 and 3 cm (23.7%) and the remaining seven patients had a tumor size <2 cm (18.4%) (Table 1). Tumor size seemed to influence the surgical strategy, with biopsy being significantly more commonly performed on larger tumors (*p*-value = 0.002).

### 3.4. Tumor Size and Primary Symptoms

The relationship between the tumor size and primary symptoms was further analyzed and is summarized in Table 2.

There was no significant relationship between tumor size and primary symptoms (*p*-value > 0.05).

### 3.5. Trends in Surgical Strategy

In 1996 and 1997, biopsy of pineal germinoma was carried out slightly more frequently than resection (nine biopsies vs. six resections), with a decreasing tendency in biopsy until 1999, and an increasing tendency of resection between 1997 and 2001. After 2001, resection and biopsy rates were used with nearly equal frequency, and biopsy has been slightly favored since 2003.

### 3.6. Postoperative Neurological Status and Complications

Due to incomplete documentation regarding postoperative neurological status and complications, 20/57 (35.1%) patients in the resection group and 10/54 (18.5%) patients in the biopsy group were excluded from the statistical analysis.

Postoperative neurological complications were reported in 16/37 (43.2%) patients after resection, and in 5/44 (11.4%) after biopsy, with total combined complication rate of 21/81 (25.9%) (Table 3). There was a significantly higher proportion of postoperative complications observed in the resection group than in the biopsy group in the documented patients (*p*-value = 0.008).

Two of the five patients in the biopsy group who developed postoperative complications underwent a stereotactic biopsy, and the other three patients underwent an endoscopic biopsy. Of the 16 patients in resection group with postoperative complications, 6 underwent partial resection, 4 subtotal, and 6 total resection.

As the vast majority of the patients underwent either a resection by an infratentorial-supracerebellar approach or a stereotactic biopsy, statistical analysis of the complication rates with regard to the approach chosen within the resection and biopsy groups respectively was not feasible due to high differences in case numbers.

## 4. Discussion

We analyzed the perioperative course in relation to the type of surgery for all patients diagnosed and treated for germinoma of the pineal region within the SIOP CNS GCT 96 study. These initial tumor-targeted surgeries were performed between 1996 and 2005 in 14 European countries, reflecting treatment in numerous neurosurgical units. The neurosurgical protocol guidelines, although highlighting the need to obtain tissue in marker-negative tumors, did not clearly specify the type of surgical approach that should be employed. For this reason, the reported data reflect the current practices for the initial neurosurgical approach in these patients, who commonly present for diagnosis at the neurosurgical unit with acute hydrocephalus.

The rate of postoperative complications/worsening was significantly higher in the group that underwent a debulking procedure than in those who underwent biopsy-only interventions, the majority of which were performed using a stereotactic or an endoscopic technique (*p* = 0.008).

Historically, the neurosurgical approach to these tumors was limited to treating the associated hydrocephalus [44] due to the technical difficulties in the surgical approach to tumors in the pineal region, the majority being germinoma on histological examination, and their excellent response to radiotherapy. Tumor removal was initially confined to selected centers of expertise that could show that resection was feasible with acceptable morbidity [33,35,45,46,47,48,49].

Over the last few decades, reports have shown that using a stereotactic biopsy approach as the initial diagnostic procedure is safe and reliable [50,51,52], an observation that has been reproduced in multicenter studies [28,52]. However, there are no systemic studies that statistically compared primary resection and biopsy on solitary pineal germinoma. Even without an underlying statistical analysis, the current existing literature slightly recommends biopsy over primary resection of pineal germinoma based on the high radio- and chemosensitivity of germinomas. Furthermore, many studies recommend primary resection for solitary pineal tumors due to possible differential diagnosis tumors, which require radical resection, such as pineocytoma, teratoma, meningioma, epidermoid, etc. [36].

With obstructive hydrocephalus being present in the majority of patients, and with endoscopic ventriculocisternostomy becoming favored as the treatment of choice, combining this procedure with a transventricular endoscopically guided biopsy became increasingly used [40,42,53,54,55,56,57,58,59]. Within the SIOP CNS GCT 96 study, NGGCTs made up 42% of tumors occurring in the pineal location. With endoscopic third ventriculostomy as the favored technique for treating the associated hydrocephalus, this approach offers the opportunity for sampling CSF for markers while obtaining a biopsy specimen within the same intervention, and has a reported rate of complication between 0 and 21.7% [40,59]. If serum markers are not be available at the time of an urgently needed intervention, and with CSF markers not yet having been performed, the surgeon would need to balance the risks of performing an ultimately redundant biopsy and the benefits of potentially avoiding the need for a second procedure.

The diagnostic yield and accuracy are generally deemed acceptable with both stereotactic and endoscopic techniques [27,40,50,52,55,56]. Nevertheless, tissue diagnosis may be less accurate with endoscopic than with stereotactic procedures (81.1% versus 93.7%), though without reaching statistical significance (*p* > 0.05) [52]. Thus, although the neuroendoscopic approach seems to be the best tool for managing hydrocephalus, stereotactic biopsies may represent the best way to obtain a tissue diagnosis with accuracy and low morbidity [52].

Conversely, an attempt at resection is currently deemed appropriate for most non-GCT pineal tumors such as glioma, pineal parenchymal tumor, CNS embryonal tumor, and ependymoma.

In a series of patients treated from the 1990s on, complication rates for pineal tumor surgery were about 0–2% for mortality, 0–5% for major morbidity, and 0–24% for minor morbidity for such entities [60,61,62,63].

Most surgical series of resection of pineal tumors of various histologies with sufficient detail on postoperative follow-up report on the experience of single institutions, often even single surgeons [64]. Postoperative morbidity of resective procedures in recent monoinstitutional series ranges between 19% and 22% [33,35,41,49,62,63,65,66,67], with up to 45.6% adverse events in the period of 30 days after surgery [68]. The finding of a 43% complication rate after resection within the SIOP CNS GCT 96 trial could be related to its multicenter/multisurgeon database. An additional factor to take into account when comparing our results with those of other series is the infiltrative nature of germinomas compared with other histological entities encountered in the pineal region. Most of the series pool these heterogeneous pathologies when reporting on complications.

Advanced neuroimaging combined with clinical information can contribute to the prediction of the tumor type and guide decision making. This may not only impact the eventual surgical approach used, but, more importantly, the management guidelines for the working diagnosis that determine the need for resection as part of treatment versus valid histological sampling only.

Whereas study protocols in Europe and North America increasingly relied on AFP and HCG to diagnose and stratify GCT, obviating the need for histological confirmation in cases with typical radiology and elevated markers, and mandating histology for marker-negative cases only, most Japanese and other East Asian groups continue to favor primary extensive resections in pineal tumors. Due to a much higher incidence of CNS GCTs in these regions, this approach was supported by documenting the histological heterogeneity and high percentage of mixed histology GCTs, thus highlighting the risk of a sampling error with a small biopsy specimen [35].

Within the SIOP CNS GCT 96 study, of the 113 patients with unifocal pineal germinomas, 1/54 initially diagnosed with a biopsy had a local relapse with a diagnosis of the NGGCT subtype yolk sac tumor (YST) [43]. One could argue that a YST component had been missed in the biopsy specimen of the initially marker-negative tumor (i.e., alpha-fetoprotein (AFP) below 25 ng/mL and human chorionic gonadotropin (hCG) below 50 UI/L in both compartments).

Within the cohort of the Japanese intracranial GCT (iGCT) Consortium, data from 93 patients with a unifocal GCT of the pineal region were collected. Twenty-six had markers elevated above the SIOP CNS GCT 96 cut-off values, with the nongerminomatous diagnosis confirmed by surgery, except for a single case with a germinoma at biopsy and AFP of 30 ng/mL in CSF. Two patients had a YST diagnosis without markers available. Of the remaining 65 patients, biopsy-only was performed in 22 cases, and a resection in 43. Two patients undergoing resection were diagnosed as mixed germinoma-embryonal carcinoma. No information on surgical morbidity was provided for this multicenter study [69]. Therefore, a tumor-marker-based strategy has its pitfalls: it is possible to undertreat patients with a nonsecreting NGGCT component, and overtreat patients with a pure germinoma that has tumor markers above the defined thresholds. This potential pitfall must be balanced with the possible morbidity of surgery.

The high cure rates for patients with pineal germinomas using current protocols are a major impediment to assessing a potential therapeutic benefit to upfront resection for such cases, with its associated with unavoidable risks, in any potential prospective study [43]. Despite the low risk of missing a treatment-relevant tumor component in a biopsy procedure, our data support the practice of biopsy-only as the primary surgical approach to marker-negative pineal region tumors with clinical/radiological suspicion of germinoma.

The limitations of this study are its retrospective nature and the heterogeneity of the surgical techniques used for biopsy and resection procedures. This heterogeneity could at least partly be related to the shift in neurosurgical approach from a center-specific standard approach to a more widespread implementation of image-guided biopsy and endoscopic techniques.

## 5. Conclusions

With the excellent sensitivity of CNS germinoma to chemotherapy and/or radiation, the neurosurgical role for germinoma needs to be very carefully evaluated. With limited evidence for the benefit of an upfront debulking procedure, biopsy-only of localized suspected germinoma in the pineal region should be favored over primary resection due to the significantly lower risk of surgical postoperative complications.

## Figures and Tables

**Table 1:** Tumor size and biopsy/resection. Biopsy was the favored operative choice in larger tumors. Using the chi-square test, a *p*-value of 0.002 was measured.

		Biopsy/Resection		Total	
		Resection	Biopsy		*p*-Value (Chi-Square Test)
Tumor size	<2 cm	7	7	14	
	2–3 cm	26	9	35	
	>3 cm	10	22	32	
Total		43	38	81	0.002

**Table 2:** Relationship between tumor size and primary symptoms. No significant relationship was found between primary symptoms and tumor size.

	Tumor Size < 2 cm		Tumor Size 2–3 cm		Tumor Size > 3 cm		*p*-Value (Chi Square Test)
	Biopsy	Resection	Biopsy	Resection	Biopsy	Resection	
Headache	6	5	9	22	20	8	0.090
Hydrocephalus	5	6	8	23	18	9	0.068
Parinaud syndrome	2	6	4	11	11	7	0.064
Papilledema	5	0	3	9	8	5	0.057
Double vision	4	6	5	17	11	3	0.050

**Table 3:** Postoperative complications (biopsy vs. resection). A total of 37/57 patients in resection group and 44/54 patients in biopsy group had complete documentation regarding postoperative complications; the others were excluded from this statistical analysis. There was a significant lower risk of postoperative complications in the biopsy group of the documented patients (*p*-value = 0.008).

			Biopsy/Resection		Total	
			Resection	Biopsy		*p*-Value (Fisher Exact Test)
Postoperative complications	No complications		21	39	60	
	Postop complications		16	5	21	*p* = 0.008
		Parinaud syndrome/double vision	9	3	12	
		Hydrocephalus	4	1	5	
		Others	3	1	4	
Total			37	44	81	

## Data Availability

The data presented in this study are available within the article.
